# From GWAS to new biology and treatments in CAD

**DOI:** 10.18632/aging.101891

**Published:** 2019-03-25

**Authors:** Peter D. Jones, Tom R. Webb

**Affiliations:** 1 Department of Cardiovascular Sciences and National Institute for Health Research Leicester Cardiovascular Biomedical Research Centre, Glenfield Hospital, University of Leicester, Leicester, United Kingdom

**Keywords:** atherosclerosis, coronary artery disease, JCAD

Coronary artery disease (CAD), which remains a leading cause of mortality worldwide [1], is caused by the development of atherosclerotic plaques in the arterial wall. Disease risk is influenced by environmental and lifestyle factors as well as having a significant genetic component. Over the last decade, genome-wide association studies (GWAS) have revealed the chromosomal loci contributing to increased CAD susceptibility, with the most recent investigation linking more than 300 genetic variants to disease [2]. A key characteristic of these loci is that most do not work through traditional CAD risk factors and current treatment targets such as plasma LDL-cholesterol levels or blood pressure. Importantly, for the majority of loci the underlying mechanism is unknown suggesting that our knowledge of disease pathogenesis is far from complete. CAD loci therefore offer a resource for the understanding of the molecular pathways and biological processes driving disease and potential identification of therapeutic targets.

In a recent study [3] we investigated *JCAD* (previously *KIAA1462*), a novel gene at the 10p11.23 CAD locus [4]. We demonstrated that JCAD mediates several CAD relevant endothelial cell phenotypes including proliferation, migration, apoptosis and adhesion molecule expression and inflammatory cell recruitment. We went on to show that JCAD interacts with the protein kinase LATS2 and functions as a new negative regulator of the Hippo signaling pathway to increase activity of the transcriptional effector YAP and downstream gene expression. We examined human gene expression data to show that CAD risk genotype associated with increased *JCAD* expression and relate *JCAD* expression modules to endothelial cell phenotype and YAP, supporting our findings that *JCAD* regulates endothelial cell function via YAP.

*RHOA*, which encodes the Rho GTPase RhoA, a negative regulator of the Hippo pathway, has also been linked to CAD in a recent GWAS [2]. RhoA promotes YAP activity in response to various stimuli including cell confluency, mechanotransduction and G-protein coupled receptor signaling. We used a Rho-kinase inhibitor to reduce RhoA activity and the thrombin agonist TRAP6 to activate RhoA and investigated their regulatory effects on YAP following *JCAD* knockdown [3]. In the absence of JCAD we found that TRAP6 activation of YAP was lost while Rho-kinase inhibition had no additive effect on YAP activation. These results suggest that JCAD is in the same part of the Hippo regulatory cascade as RhoA.

Atherosclerotic plaques develop at sites of low or oscillatory shear stress, while laminar shear stress is atheroprotective. Notably, recent work has identified RhoA and YAP as key mediators of these effects [5,6]. Atheroprotective laminar flow activates endothelial Intergrin proteins thereby inhibiting RhoA activity causing an increase in YAP phosphorylation and its exclusion from the nucleus. RhoA is activated under disturbed flow leading to increased YAP nuclear localisation with the resulting changes in gene expression causing pro-atherogenic endothelial cell phenotypes. Our results implicate JCAD, LATS2 and the Hippo pathway as the mediators of this process [3].

The RhoA-JCAD-Hippo-YAP pathway is a promising new drug target for the treatment of CAD. RhoA is a modulator of the non-lipid lowering atheroprotective effects of statins and statin treatment of endothelial cells inhibits YAP activity [5,6]. In addition, the Rho kinase inhibitor Fasudil, which has been approved for therapeutic use in Japan and China for treatment of cerebral vasoplasm [7], reduces atherosclerosis in mice [8].

In summary, GWAS have identified genetic variants in *RHOA* and *JCAD* associated with increased risk of CAD and recent mechanistic studies have shown that these two genes regulate the Hippo signalling pathway and YAP activity to control endothelial cell function in atherosclerosis. Targeting this pathway via new or existing inhibitors such as Fasudil might provide a new treatment opportunity for CAD.
